# Boosting Chemodynamic Therapy by the Synergistic Effect of Co-Catalyze and Photothermal Effect Triggered by the Second Near-Infrared Light

**DOI:** 10.1007/s40820-020-00516-z

**Published:** 2020-09-02

**Authors:** Songtao Zhang, Longhai Jin, Jianhua Liu, Yang Liu, Tianqi Zhang, Ying Zhao, Na Yin, Rui Niu, Xiaoqing Li, Dongzhi Xue, Shuyan Song, Yinghui Wang, Hongjie Zhang

**Affiliations:** 1 State Key Laboratory of Rare Earth Resource Utilization, Changchun Institute of Applied Chemistry (CIAC), Chinese Academy of Sciences (CAS), Changchun, 130022 People’s Republic of China; 2 University of Chinese Academy of Sciences, Beijing, 100049 People’s Republic of China; 3 Department of Chemistry, Tsinghua University, Beijing, 100084 People’s Republic of China; 4 Department of Radiology, The Second Hospital of Jilin University, Changchun, 130041 People’s Republic of China

**Keywords:** Chemodynamic therapy, *Fenton* reaction, Co-catalysis, Photothermal effect, *NIR* II biowindows

## Abstract

In spite of the *tumor* microenvironments responsive cancer therapy based on *Fenton* reaction (i.e., chemodynamic therapy, CDT) has been attracted more attentions in recent years, the limited *Fenton* reaction efficiency is the important obstacle to further application in clinic. Herein, we synthesized novel FeO/MoS_2_ nanocomposites modified by bovine serum albumin (FeO/MoS_2_-BSA) with boosted *Fenton* reaction efficiency by the synergistic effect of co-catalyze and photothermal effect of MoS_2_ nanosheets triggered by the second near-infrared (*NIR* II) light. In the *tumor* microenvironments, the MoS_2_ nanosheets not only can accelerate the conversion of Fe^3+^ ions to Fe^2+^ ions by Mo^4+^ ions on their surface to improve *Fenton* reaction efficiency, but also endow FeO/MoS_2_-BSA with good photothermal performances for photothermal-enhanced CDT and photothermal therapy (PTT). Consequently, benefiting from the synergetic-enhanced CDT/PTT, the tumors are eradicated completely in vivo. This work provides innovative synergistic strategy for constructing nanocomposites for highly efficient CDT.

**Electronic supplementary material:**

The online version of this article (10.1007/s40820-020-00516-z) contains supplementary material, which is available to authorized users.

## Highlights

- The MoS_2_ nanosheets served as co-catalyst could reduce Fe^3+^ ions with lower *Fenton* reaction activity into the highly reactive Fe^2+^ ions, thereby boosting the production of hydroxyl radical (•OH) for high efficiency chemodynamic therapy (CDT).
- The photothermal effect of MoS_2_ nanosheets motivated by second near-infrared light could further improve the treatment effectiveness by synergetic photothermal-enhanced CDT and photothermal therapy.

**Electronic supplementary material:**

The online version of this article (10.1007/s40820-020-00516-z) contains supplementary material, which is available to authorized users.

## Graphical abstract

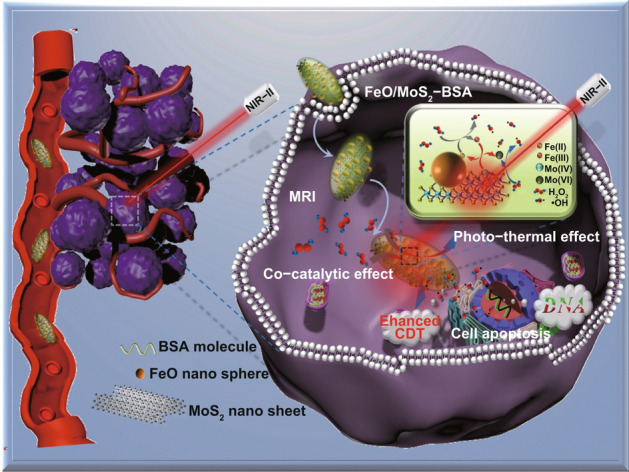

## Introduction

Nowadays, cancer is an illness which threatens the human health owing to its high mortality rate and recurrence rate [1, 2]. Several treatment strategies have been applied in clinic, such as surgery [3], radiotherapy [4], and chemotherapy [5, 6], but  they still suffer from limited treatment effect and significant side effects. Recently, chemodynamic therapy (CDT) based on *Fenton* and *Fenton*-like reactions (denoted as generating highly oxidative hydroxyl radicals from hydrogen peroxide by ferrous or non-ferrous ions to remove organic pollutant) has drawn more attentions because it can generate the highly toxic reactive oxygen species [hydroxyl radicals (•OH)] in the *tumor* lesion area, resulting in high specificity and low side effects [7–13]. However, the weak acid and low transformation efficiency of Fe^3+^ to active Fe^2+^ in the *tumor* microenvironments lower the *Fenton* reaction efficacy, which leads to unsatisfactory therapy effect of CDT [14, 15]. Therefore, it is urgent to develop new strategy to improve the *Fenton* reaction efficiency in the *tumor* microenvironments.

Recently, some promising strategies have been used to improve the treatment effect of CDT. For instance, Bu and coworker take advantage of photothermal effect to enhance the *Fenton* reactions efficiency, and obtain the good treatment effect of combinatorial CDT/PTT [7]. Our group has successfully utilized the light, ultrasound, and near-infrared II (*NIR* II) light-triggered photothermal effect to assist the *Fenton* reaction and improve the treatment effect. In addition to these methods that depend on the external stimulus to enhance the *Fenton* reactions efficiency [16, 17], the strategy that introduces a catalyst to accelerate the rate-controlling step of Fe^3+^/Fe^2+^ conversion has been attracted much more attentions recently in the field of treating persistent pollutants [18, 19]. However, the research on improving the therapeutic efficacy of CDT with this co-catalytic strategy is rarely reported till now [20, 21]. If the catalyst also has good photothermal performance response to *NIR* II light for PTT, the improved therapeutic efficacy of CDT will be achieved through the synergistic effect.

Two-dimensional (2D) molybdenum disulfide (MoS_2_) nanosheets have been used as photothermal agents owing to strong *NIR* absorbance and high photothermal conversion efficiency [22–25]. Meanwhile, MoS_2_ nanosheets have the peroxidase-mimic capability, making them catalyze decomposition of H_2_O_2_ to generate •OH [10, 26, 27]. Importantly, the active Mo^4+^ ions on the surface of MoS_2_ nanosheets can reduce Fe^3+^ ions into Fe^2+^ ions, accelerating the conversion of Fe^3+^ to Fe^2+^ [19]. Therefore, MoS_2_ nanosheets are good candidates to improve the therapeutic efficacy of CDT through the synergistic effect of photothermal effect/co-catalysis.

**Scheme 1:**
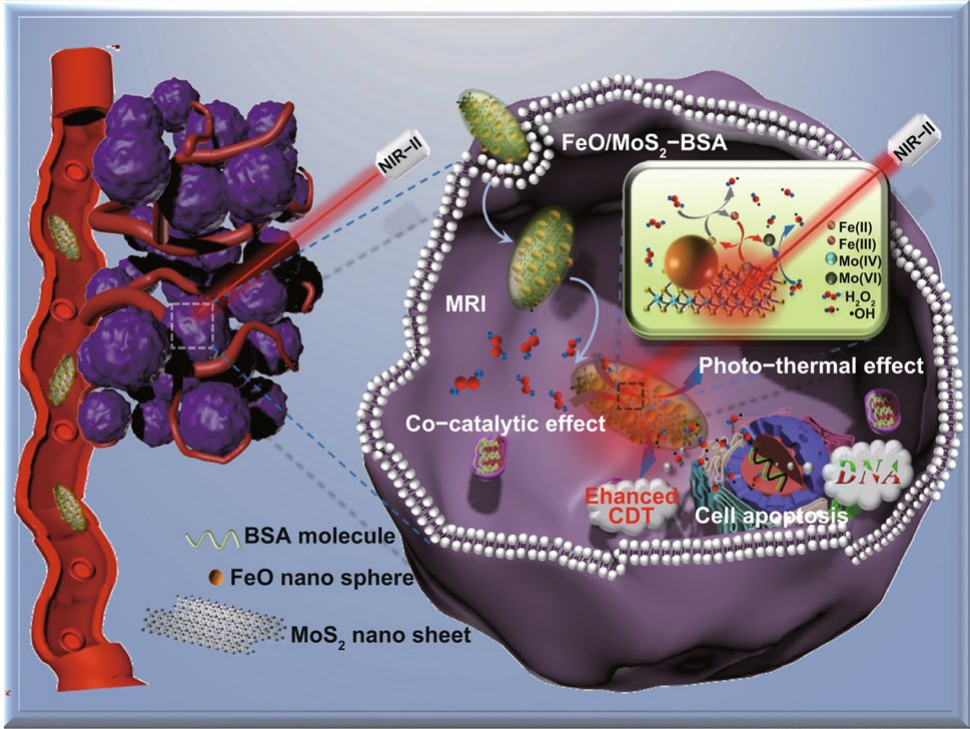
Schematic presentation of FeO/MoS_2_-BSA for MRI and synergetic enhanced CDT/PTT

Building from these ideas, herein, we design and synthesize novel FeO/MoS_2_ nanocomposites modified by bovine serum albumin (FeO/MoS_2_-BSA) for *magnetic* resonance *imaging* guided highly efficient CDT (Scheme 1). FeO/MoS_2_-BSA exhibits higher production efficiency of •OH compared to that of FeO and MoS_2_ individually, demonstrating that the co-catalysis strategy can improve the *Fenton* reaction efficacy. Moreover, owing to the absorption ability of MoS_2_ in *NIR* II region (1000–1350 nm), the photothermal (PT) effect of FeO/MoS_2_-BSA triggered by *NIR* II light (1064 nm) has been first employed to further improve the production efficiency of •OH. As the results, not only the cancer cells could be effectively killed by synergetic enhanced CDT and photothermal therapy (PTT), but also the tumors are eliminated completely in vivo experiments, indicating their highly efficient therapeutic efficiency in vitro and in vivo. In addition, the good *magnetic* properties of FeO endows FeO/MoS_2_-BSA with great potential as contrast agents for MRI [28, 29]. This work thus presents a synergistic strategy of *NIR* II light motivate photothermal effect [30–33] and co-catalysis to construct nanotheranostic agents with high potency and low side effects.

## Materials and Methods

### Materials

Ammonium molybdate, thiourea, NAC, iron(III) acetylacetonate (99.9%), trioctylamine (98%), oleic acid (90%), nitrosonium tetrafluoroborate (NOBF_4_), bovine serum albumin (BSA), p-phthalic acid (99%) were purchased from Aladdin company. Calcein acetoxymethyl ester (Calcein AM), propidium iodide (PI), 2,7-dichlorofluorescin diacetate were obtained from Sigma-Aldrich (MO, USA). The CCK-8 was purchased from Changchun Sanbang Pharmaceutical Technology Co (Changchun, China). Other chemicals were purchased from Beijing Chemical Reagent Company.

### Preparation of FeO/MoS_2_-BSA Nanocomposites

#### Preparation of MoS_2_ Nanosheets

The large-scale MoS_2_ nanosheets were prepared following to the previous literature [23]: ammonium molybdate (50 mg, 0.25 mmol) and NAC (50 mg, 0.3 mmol) were dissolved in ultrapure water (30 mL) in ice-water bath under stirring vigorously. Then, thiourea (40 mg, 0.5 mmol) was poured into the precursor solution under vigorous stirring for 30 min. The mixture was added into a 40-mL Teflon-lined stainless steel autoclave at 200 °C for 8 h. After cooling to room temperature, collected by centrifugation at 8000 r min^−1^ for 5 min and washed with water for three times. The small MoS_2_ nanosheets were obtained large-scale nanosheets under ultraphonic effect of ultrasonic cell disruptor for 1 h, and then collect the upper suspension after centrifugation and re-dispersed in 10 mL water.

#### Preparation of FeO Nanoparticles

The FeO nanoparticles were synthesized by the *thermal* decomposition method [34]. The iron(III) acetylacetonate (500 mg), oleic acid (0.52 mL), and trioctylamine (20 mL) were sequentially added into three-necked flask and heated at 150 °C for 30 min to remove water under argon gas protection, then slowly heated to 270 °C and kept for 10 min (rise rate: 2 °C min^−1^). After cooling to room temperature, FeO nanoparticles were collected by centrifugation at 8000 rpm for 10 min, and washed with cyclohexane and ethyl alcohol mixed solution for three times. The hydrophilic FeO nanoparticles were obtained by ligand-exchange strategy as follows [35]: 5 mL of FeO nanoparticles were dispersed in hexane (∼5 mg mL^−1^), and then 5 mL of dichloromethane solution of NOBF_4_ (0.01 M) was added into solution at room temperature under stirring vigorously for 10 min. After that, the hydrophilic FeO nanoparticles were obtained by centrifugation at 2000 r min^−1^ for 5 min and washed with toluene and hexane (1:1 by volume) for two times.

#### Preparation of FeO/MoS_2_-BSA Nanocomposites

The FeO nanoparticles solution (1 mL, 1000 ppm) was dropped into 10 mL of MoS_2_ nanosheets with mass concentration of Mo (200 ppm), respectively, under stirring vigorously for 20 min, and then BSA solution (1 mL, 5 mg mL^−1^) was added into above mixed solution individually for 1 h. The nanocomposites were collected by centrifugation at 8000 r min^−1^ for 10 min and washed with water for two times.

### Characterization

Powder X-ray diffraction (XRD) was tested on a Bruker D8 Focus powder X-ray diffraction with Cu Kα radiation (*λ* = 1.5418 Å) at 40 kV and 40 mA. Inductively coupled plasma (ICP) analyses were obtained from Varian Liberty 200 spectrophotometer to determine the contents. The UV−vis−*NIR* spectra were recorded on spectrometer (SHIMADZU, UV-3600). Infrared *thermal* *imaging* camera (FLIR T420, Fluke, USA). Transmission electron microscopic (TEM) images were obtained from a TECNAI G2 high resolution transmission electron microscope, operating at 200 kV. X-ray photoelectron spectroscopy (XPS) spectra were performed on an ESCALAB-MKII 250 photoelectron spectrometer (VG Co.) with Al Kα X-ray radiation as the X-ray source for excitation. Discovery MR750w, GE, America. Bruker Avance III (9.4 T, 400 MHz) NMR spectrometer for *magnetic* resonance *imaging*. *Fluorescence* spectrometer (F-4800) for detection of PTA.

### Detection of Extracellular •OH

Series of sample solutions were prepared by adding different samples into total reaction volume of 2 mL PBS buffer solution (pH 6.0) containing H_2_O_2_ (0.5 mM) and PTA as *fluorescence* probe (25 mg L^−1^) for *fluorescence* intensity detection (EX: 315 nm, EM: 425 nm) with time. Samples are as follows: FeO/MoS_2_-BSA, MoS_2_ nanosheets, FeO nanoparticles.

### Photothermal Effect and Thermal Stability of FeO/MoS_2_-BSA Nanocomposites

The temperature of FeO/MoS_2_-BSA nanocomposites solution with different mass concentrations (0, 100, 200, and 400 μg mL^−1^) were measured by a thermocouple probe every minute for 10 min under the 1064 nm laser irradiation (1.0 W cm^−2^) and 808 nm laser irradiation (0.3 W cm^−2^). The temperature of FeO/MoS_2_-BSA nanocomposites solution (200 μg mL^−1^) were measured by a thermocouple probe every minute for 10 min under the 1064 nm laser irradiation with different laser power (0.3, 0.5, 0.75, and 1.0 W cm^−2^). As for the *thermal* stability, the FeO/MoS_2_-BSA nanocomposites solution (200 μg mL^−1^) was irradiated with 1064 nm laser (0.75 W cm^−2^) in quartz cuvette for 10 and 60 min.

### MRI *imaging* Property

The *T*_2_-weighted MR *imaging* of FeO/MoS_2_-BSA nanocomposites with mole concentration ratio of Fe (0, 0.03, 0.06, 0.12, 0.25, and 0.50 mM) were tested on the 3.0 T clinical scanner (Discovery MR750w, GE, America). The *T*_2_ relaxation time of FeO/MoS_2_-BSA nanocomposites solutions (${\rm V}_{{{\rm H}_2}{\rm O}}:{\rm V}_{{{\rm D}_2}{\rm O}}$ = 1:1) with different mole concentrations of Fe was measured by Bruker Avance III (9.4 T, 400 MHz) NMR spectrometer.

### Cell Experiment

#### Cell Culture

Human cervical adenocarcinoma epithelial cells (HeLa) were cultured with regular growth medium containing high glucose DMEM at 37 °C in a 5% CO_2_ environment. Cell culture media with different pH were adjusted by HCl.

#### Cytotoxicity Measurement of FeO/MoS_2_-BSA Nanocomposites

HeLa cells were seeded into 96-well plates for 24 h (37 °C, 5% CO_2_). Then, FeO/MoS_2_–BSA nanocomposites (0, 50, 100, 200, and 400 μg mL^−1^) were added into 96-well plates for 24 h. Then, the HeLa cells were washed with PBS for 2 times. Then CCK-8 solution (100 μL) was added and maintained for 3 h. At last, the 96-well plates were putted into plate reader for analysis and the absorbance at 450 nm was recorded to measure the cell viability. Except for adding H_2_O_2_ and adjusting pH value of DMEM (pH = 6.5), the other experiment is same as mentioned above for cytotoxicity measurement of FeO/MoS_2_-BSA nanocomposites in stimulated TME.

#### In Vitro Photothermal-assisted CDT of FeO/MoS_2_-BSA Nanocomposites

HeLa cells were seeded in 96-well plates for 24 h. Then, FeO/MoS_2_-BSA nanocomposites solution (0, 50, 100, 200, and 400 μg mL^−1^) were added into the HeLa cells containing H_2_O_2_ (0.01 mM) for 24 h. Then, the HeLa cells were irradiated with 1064 nm laser (0.75 W cm^−2^) for 10 min. After that, the HeLa cells were washed with PBS for 2 times and incubated with CCK-8 solution (100 μL) for 3 h. At last, the 96-well plates were putted into plate reader for analysis and the absorbance at 450 nm was recorded to measure the cell viability.

#### *Detection of Intracellular•OH* by DCFH-DA

HeLa cells were seeded in six-well plate containing H_2_O_2_ for 12 h. FeO/MoS_2_-BSA with mass concentration of 200 μg mL^−1^ were added into and incubated for 4 h. After incubating with DCFH-DA (10 μM) with or without 1064 nm laser irradiation (0.75 W cm^−2^) for 5 min and after washing with PBS for two times, the *fluorescence* *imaging* of cells were monitored by confocal microscopy.

#### PI/AM Co-staining

HeLa cells were seeded in six-well plate for 12 h. FeO/MoS_2_-BSA with mass concentration of 200 μg mL^−1^ in DMEM containing H_2_O_2_ were added and incubated for 4 h. Then, cells were irradiated with or without 1064 nm laser (0.75 W cm^−2^) for 10 min. After washing with PBS for two times and co-stained with calcein AM and propidium iodide (PI) for 30 min. After washing with PBS for two times, the cellular modality of cells was monitored by *fluorescence* microscopy.

### Animal Experiment

All of the animal experiments were carried out under the NIH guidelines for the care and use of laboratory animals (NIH Publication No. 85-23 Rev. 1985) and approved by the Jilin University Animal Care and Use Committee. Kunming mices (20 g) were purchased from Laboratory Animal Center of Jilin University (Changchun, China).

#### In Vivo* Therapy*

The U14 cells were subcutaneously injected into the underarm of Kunming mice. Thirty mice bearing U14 *tumor* (≈120 mm^3^) were randomly allocated into four groups: (a) control group, (b) laser group, (c) FeO/MoS_2_-BSA nanocomposites group, (d) FeO/MoS_2_-BSA nanocomposites + laser group. The mice in the (b) and (d) group were irradiated under the 1064 nm laser for 10 min with 0.75 W cm^−2^ laser power after tail intravenous injection with equal volume of physiological saline (200 µL) and FeO/MoS_2_-BSA nanocomposites (200 µL, 400 µg mL^−1^), respectively. The same procedure was applied in c) and d) group with exception of no laser irradiation. The treatment procedure with or without 1064 nm laser irradiation was repeated four times in four days, the electronic balance and digital calipers were used to measure the body weight and *tumor* size every 2 days. The *tumor* volume was calculated by the equation (Volume = (*Tumor* Length) × (*Tumor* Width)^2^/2). The tumors were dissected out and photographed to evaluate the therapeutic effect after 2 weeks.

#### In Vivo* MRI*

The *T*_2_-weighted MRI of *tumor* bearing mice were conducted by using 3.0 T clinical scanner (Discovery MR750w, GE, America) after tail intravenous injection with FeO/MoS_2_-BSA nanocomposites (200 µL, 400 µg mL^–1^) for 0, 4, 12, and 24 h. The *T*_2_-weighted *imaging* was disposed with the following optimal parameters of instrument (TE = 104.6 ms,TR = 3000 ms, FOV = 200 × 200 mm).

#### Biodistribution of FeO/MoS_2_-BSA Nanocomposites

The *tumor*-bearing mice were necked off after intravenously injected with FeO/MoS_2_-BSA nanocomposites solution (400 μg mL^−1^, 200 μL) for 1, 7, and 14 d. Then, the heart, liver, spleen, lungs, kidneys, and tumors of mice were dissected out. After recording the weight of every tissues, 5 mL of digesting aquaregia (HNO_3_: HCl = 1: 3) was added for 48 h. The content of Mo was measured by ICP-AES.

#### TUNEL and H&E Staining

The *tumor*-bearing mice were killed on the third day, the *tumor* tissues were dissected out for hematoxylin and eosin (H&E) staining and TdT-mediated dUTP nick end labeling (TUNEL). *Tumor*-bearing mice were necked off on the 14th day, and the heart, liver, spleen, lung, kidney were dissected out and immersed in formalin and processed in paraffin for H&E staining.

## Results and Discussion

### Characterization of FeO/MoS_2_-BSA Nanocomposites

**Fig. 1:**
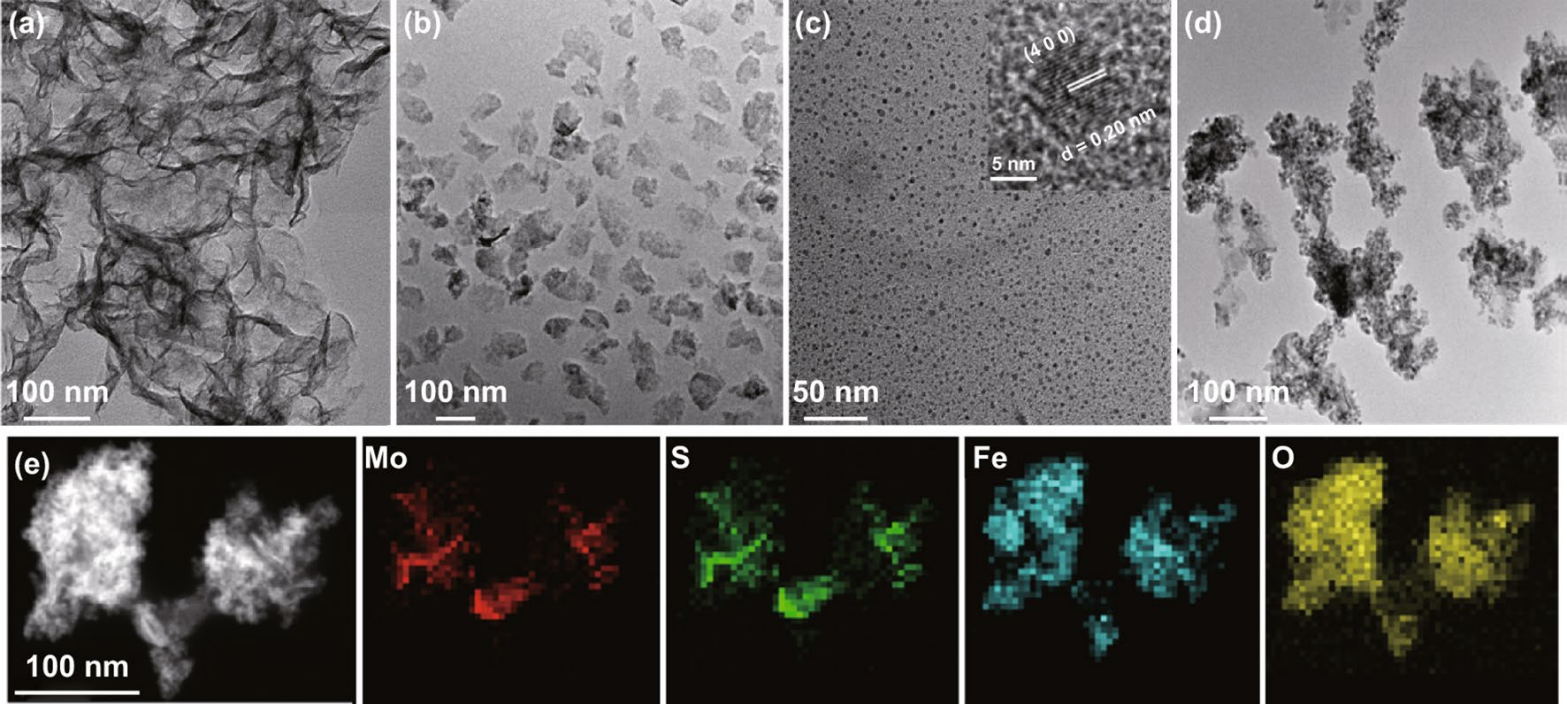
Characterization of FeO/MoS_2_-BSA nanocomposites. **a**, **b** TEM images of large and small MoS_2_ nanosheets. **c**, **d** TEM images of FeO nanoparticles and FeO/MoS_2_-BSA nanocomposites, and inset in **c** HRTEM image of FeO nanoparticle. **e** STEM image and EDS elemental mapping of FeO/MoS_2_-BSA nanocomposites

The MoS_2_ nanosheets with average size of 150 nm were obtained after sonicating the solution of large MoS_2_ sheets (> 1 μm) which is shown in Fig. 1a, b, and as shown in Fig. S1a, the X-ray diffraction (XRD) pattern can be indexed well to 2H–MoS_2_ phase (JCPDS No. 37-1492). The ligand-free FeO nanospheres were synthesized following the previous report [34], and the average diameter was determined to be 8 ± 3 nm by transmission electron microscopy which is (TEM) shown in Figs. 1c and S2. The high resolution TEM image in the inner picture of Fig. 1c shows that the lattice fringes with d-spacing of 0.20 nm corresponds to the (4 0 0) plane of FeO, which is consistent with the FeO (JCPDS No. 19-0629, Fig. S1b). Afterward, FeO/MoS_2_ nanocomposites were obtained by electrostatic interaction, and modified by bovine serum albumin (BSA) to improve their biocompatibility (Fig. S3). The TEM image in Fig. 1d illustrates that FeO nanospheres were successfully anchored on the surface of MoS_2_ nanosheets. The energy dispersive X-ray (EDX) mappings shown in Fig. 1e confirm the chemical composition of FeO/MoS_2_-BSA nanocomposites. The characteristic diffractions of FeO and MoS_2_ can be indexed in XRD pattern and selected area electron diffraction (SAED) of FeO/MoS_2_-BSA nanocomposites (Figs. S1c and S4), further demonstrating the successful synthesis of FeO/MoS_2_-BSA nanocomposites. The X-ray photoelectron spectroscopy (XPS) analysis provides more information of the composition and surface electronic states of FeO/MoS_2_-BSA nanocomposites (Fig. S5). The high-resolution Fe 2p spectrum shows two peaks at 710.5 and 724.3 eV, which were attributed to Fe^2+^. The peaks at 228, 232, and 225 eV correspond to the data reported for Mo 3d_5/2_ (Mo^4+^), Mo 3d_3/2_ (Mo^4+^), and S 2s, and two peaks at 161 and 162 eV represent the 2p_3/2_ and 2p_1/2_ of S^2−^, revealing the existence of MoS_2_ in the nanocomposites [23]. The presence of the peaks of N 1s and C 1s confirms the successful modification BSA on the surface of FeO/MoS_2_. The Fourier transform infrared spectroscopy (FT–IR) results further evidence the successful BSA functionalization (Fig. S6). The hydrodynamic diameters of FeO/MoS_2_-BSA nanocomposites in normal saline and PBS solution are 227.1 and 246.0 nm with low PDI value, respectively, indicating their good dispersity and stability in physiological environments (Fig. S7, Table S1).

### Synergetic Enhanced CDT/PTT of FeO/MoS_2_-BSA in vitro

**Fig. 2:**
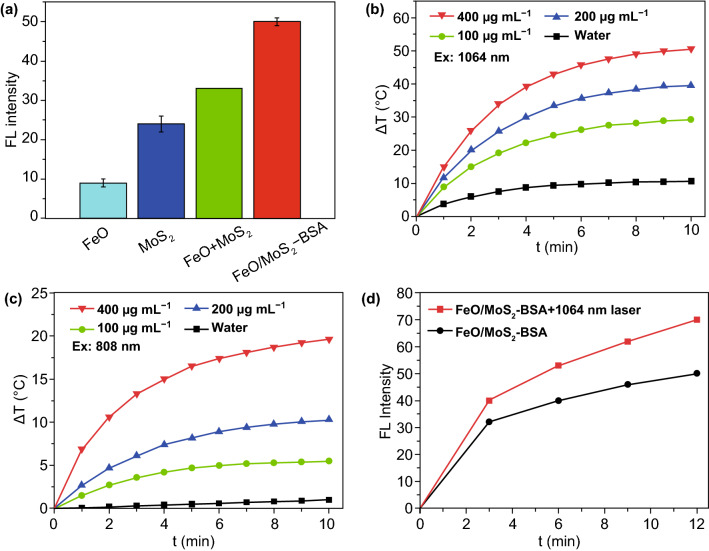
**a** •OH production capacity of FeO, MoS_2_, MoS_2_ + FeO, and FeO/MoS_2_-BSA solutions mixed with H_2_O_2_. **b**, **c** Temperature curves of different concentration FeO/MoS_2_-BSA under irradiation with 1064 nm and 808 nm laser (1 and 0.3 W cm^−2^ separately). **d** PT-enhanced •OH generation of FeO/MoS_2_-BSA nanocomposites with or without 1064 nm laser irradiation (0.75 W cm^−2^)

To evaluate the •OH production capacity of FeO/MoS_2_-BSA nanocomposites, p-phthalic acid (PTA) was choosed as *fluorescence* probe to monitor the •OH production in phosphate buffer solution (PBS) with pH value of 6.0, whose *fluorescence* emission at 425 nm enhanced as the increasing of the amount of •OH. As shown in Fig. 2a, the •OH production ability of FeO/MoS_2_-BSA is not only higher than that of FeO and MoS_2_ separately, but also the total amount of them (FeO + MoS_2_), indicating that the co-catalyst MoS_2_ can actually improve the generation efficiency of •OH. This result maybe can ascribe to the fact that the active Mo^4+^ ions on the surface of MoS_2_ nanosheets can reduce Fe^3+^ ions into Fe^2+^ ions, accelerating the conversion of Fe^3+^ to active Fe^2+^.

Moreover, FeO/MoS_2_-BSA nanocomposites exhibit stronger absorption at 808 and 1064 nm than that of FeO, indicating MoS_2_ nanosheets endow them with good photothermal property (Fig. S8). Then, we compared the photothermal effects of different concentrations of FeO/MoS_2_-BSA nanocomposites exposed to 808 and 1064 nm laser with maximum permissible exposure (MPE, 0.3 W cm^−2^ for 808 nm and 1 W cm^−2^ for 1064 nm), respectively. As shown in Fig. 2b, c, the temperature of FeO/MoS_2_-BSA nanocomposites (200 μg mL^−1^) could increase to 57 °C after irradiation with 1064 nm laser for 10 min. In contrast, it just reaches to 30 °C under illumination with 808 nm laser in the same conditions. In fact, even irradiation with 1064 nm laser at 0.75 W cm^−2^ (lower than MPE: 1 W cm^−2^), FeO/MoS_2_-BSA nanocomposites show good photothermal performances (Fig. S9). The photothermal conversion efficiency was calculated to be 56% for 1064 nm (Figs. S10 and S11), demonstrating the outstanding photothermal performance of FeO/MoS_2_-BSA nanocomposites responsive to *NIR* II light. As shown in Fig. S12, there is no absorption decrease in FeO/MoS_2_-BSA after irradiation for 1 h, revealing that the FeO/MoS_2_-BSA possess good photothermal stability. Importantly, the •OH production capacity of FeO/MoS_2_-BSA nanocomposites was effectively enhanced after irradiation with 1064 nm laser for 12 min (Figs. 2d and S13), which indicates that the photothermal effect of FeO/MoS_2_-BSA nanocomposites triggered by *NIR* II light could not only cause cancer cells death for PTT, but also achieve highly efficient CDT.

**Fig. 3:**
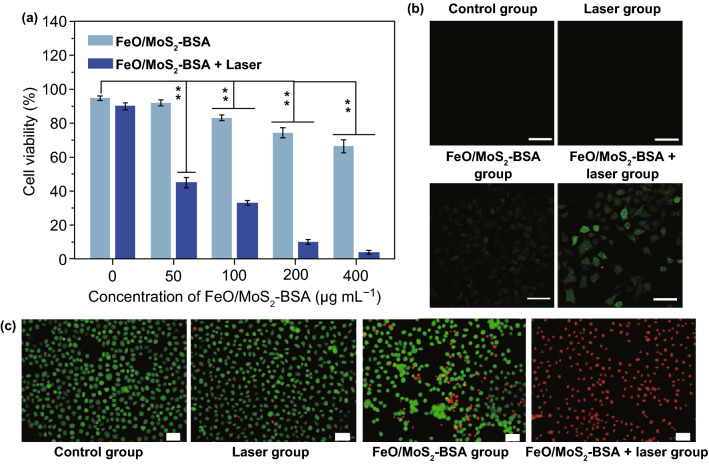
**a** Cell viability of Hela cells with different treatments (Data are means ± SD; *N* = 3). **b** Confocal *fluorescence* images of Hela cells treated with FeO/MoS_2_-BSA nanocomposites and DCFH-DA with or without 1064 nm irradiation (0.75 W cm^−2^). **c** *fluorescence* images of Hela cells co-stained with Calcein AM and PI after various treatments (**P < 0.01). Scale bar: 50 µm

For further application in vivo, the potential cytotoxicity of FeO/MoS_2_-BSA nanocomposites was evaluated by a standard Cell Counting Kit-8 (CCk-8) assay. As shown in Fig. 3a, after incubated with different concentrations of FeO/MoS_2_-BSA nanocomposites for 24 h, no significant cytotoxicity for HeLa cells was observed, showing their good biocompatibility (Fig. S14). However, the cell viability decreased with the concentration increase in FeO/MoS_2_-BSA nanocomposites under stimulated *tumor* microenvironments (100 μM H_2_O_2_), which could be attributed to the DNA damage of HeLa cells caused by •OH production through *Fenton* reaction. After irradiation with 1064 nm laser for 10 min, the cell viability is no more than 10%, showing the highly efficient antitumor efficacy of combinatorial PTT and PT-enhanced CDT. To confirm the generation of active •OH in cells, 2,7-dichlorofluorescein diacetate (DCFH-DA) was used as *fluorescence* probe for tracking ROS production, which produces green *fluorescence* triggered by •OH. As shown in Fig. 3b, compared to the control and laser groups, the weak green emission was observed after treated with FeO/MoS_2_-BSA nanocomposites, demonstrating the generation •OH in HeLa cells by Fe^2+^ and overpressed H_2_O_2_ through *Fenton* reaction. Moreover, FeO/MoS_2_-BSA+ 1064 nm laser group shows obviously enhanced green *fluorescence*, indicating that the photothermal effects of FeO/MoS_2_-BSA nanocomposites triggered by *NIR* II laser illumination can significantly improve the generation efficiency of •OH. The good treatment effect of FeO/MoS_2_-BSA nanocomposites on HeLa cells was further proved by calcein AM and propidium iodide (PI) staining. As shown in Fig. 3c, the control and laser groups show the strong green *fluorescence* (live cells) and ignorable red *fluorescence* (dead cells), but the FeO/MoS_2_-BSA nanocomposites group shows enhanced red *fluorescence*, indicating the destruction of HeLa cells by •OH. The stronger red *fluorescence* was observed after irradiation with 1064 nm laser for 10 min, revealing the good antitumor effect of FeO/MoS_2_-BSA nanocomposites by PTT and PT-enhanced CDT. All these results show that FeO/MoS_2_-BSA nanocomposites have good potential for effective PT-enhanced CDT/PTT synergistic therapy.

### Synergetic Enhanced CDT/PTT Effect of FeO/MoS_2_-BSA in vivo

**Fig. 4:**
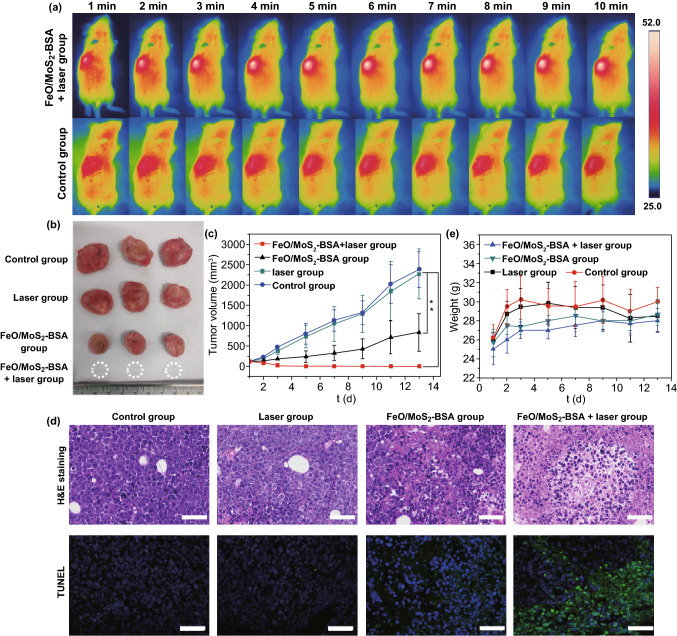
**a** IR *thermal* images of Kunming *tumor*-bearing mice with tail vein injection of normal saline and FeO/MoS_2_-BSA nanocomposites (200 µL, 400 µg mL^−1^) under 1064 nm laser irradiation (0.75 W cm^−2^) in every minute for 10 min. **b** *Tumor* photos harvested from the mices after various treatment at 14th day. **c** *Tumor* volume growth on mice measured after different treatments every 2 days for 2 weeks (*n* = 6, **P < 0.01). **d** H&E and TUNEL staining of *tumor* sections harvested from the mice after different treatments at third day (scale bars: 50 μm). **e** *Tumor* weight growth on mice measured after different treatments every 2 days within 2 weeks

**Fig. 5:**
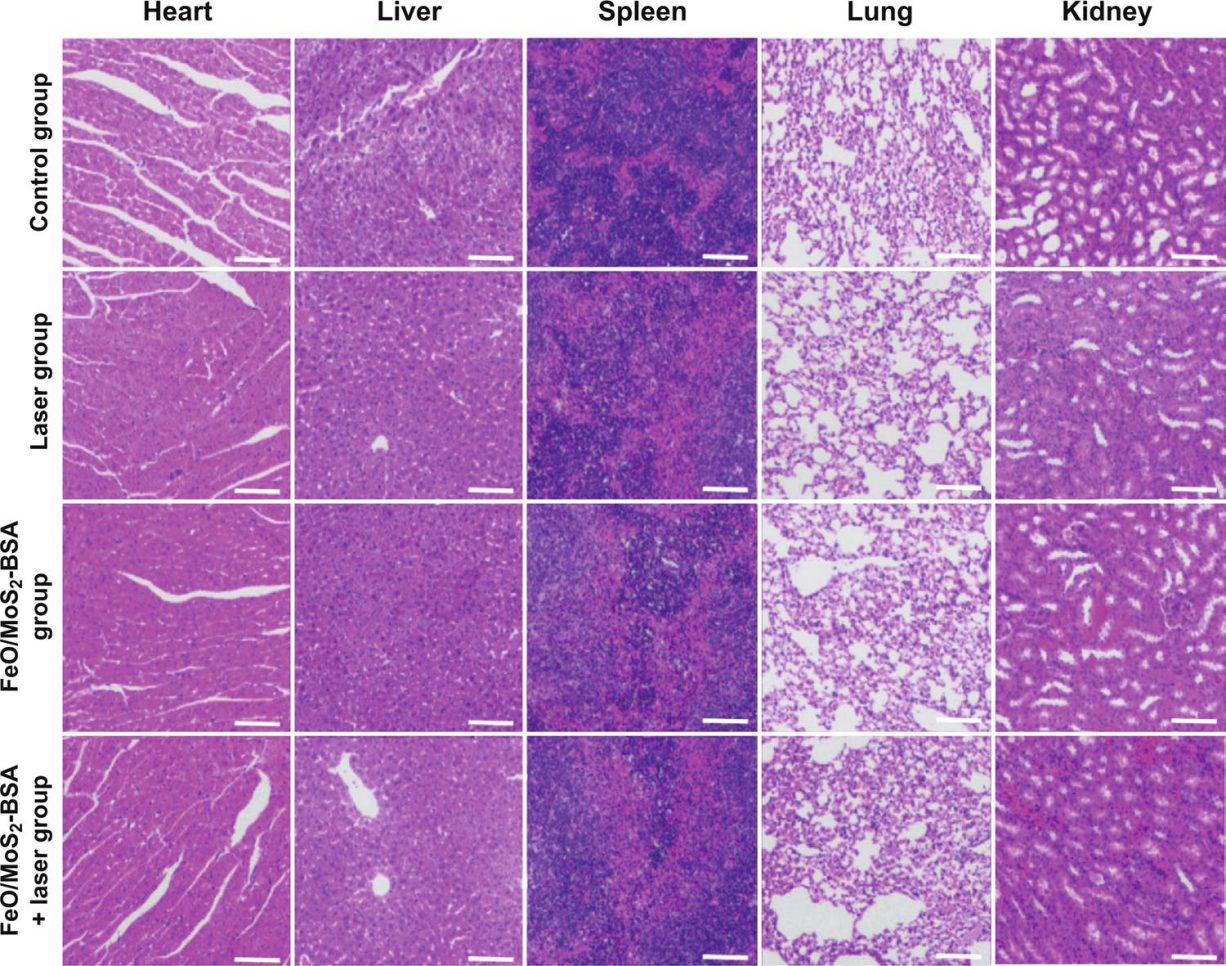
H&E staining of organs (heart, liver, spleen, lung, and kidney) harvested from the mices after different treatments at 14th day (scale bars: 100 μm)

Encouraged by the good antitumor effect in vitro, we further investigated the tumor inhibiting efficacy of FeO/MoS_2_-BSA nanocomposites on U14 *tumor* xenograft model. The photothermal performances of FeO/MoS_2_-BSA nanocomposites in vivo were confirmed by IR *thermal* camera. As shown in Figs. 4a and S15, compared to the control group, the temperature of *tumor* site rapidly increased to 52 °C under the 1064 nm laser irradiation for 10 min after intravenous injection with FeO/MoS_2_-BSA nanocomposites, demonstrating that FeO/MoS_2_-BSA nanocomposites could be served as good photothermal agent responsive to the *NIR* II light for achieving highly efficient PTT and promoting *Fenton* reaction efficiency. Then, the mice bearing *tumor* were randomized into four groups: (a) control group, (b) laser group, (c) FeO/MoS_2_-BSA nanocomposites group, and (d) FeO/MoS_2_-BSA nanocomposites + laser group. The *tumor* volume and weight of mices were recorded every 2 days. As shown in Fig. 4b, c, the *tumor* growth treated with FeO/MoS_2_-BSA nanocomposites is obviously slower than those of control group and laser group, which could be ascribed to the good co-catalytic effect of MoS_2_ and FeO for CDT. In contrast, it was found that the tumors treated with FeO/MoS_2_-BSA nanocomposites and exposed to 1064 nm laser were thoroughly ablated on the 5th day without recurrence within 2 weeks, indicating the excellent anticancer efficacy of synergistic PT-enhanced CDT/PTT. Such result was further proved by the histological analysis of *tumor* tissues with hematoxylineosin (H&E) and TdT-mediated dUTP-biotin nick and labeling staining (TUNEL). As shown in Fig. 4d, in contrast with control group and laser group, the *tumor* tissues of FeO/MoS_2_-BSA nanocomposites group and FeO/MoS_2_-BSA nanocomposites + laser group showed obviously necrosis and apoptosis, implying the treatment efficacy of CDT and PT-enhanced CDT/PTT. In additions, the weight of mice in all groups did not decrease over the duration of treatment, and the H&E staining results of major organs showed no visible damage compared to the control group, further certifying the low toxicity of FeO/MoS_2_-BSA nanocomposites to mice (Figs. 4e and 5). For further investigating the biocompatibility of FeO/MoS_2_-BSA nanocomposite, the blood biochemistry assay after intravenous injection with FeO/MoS_2_-BSA nanocomposites for 30 d was conducted. As shown in Fig. S16, there is no remarkable variation of blood index compared with control group, demonstrating good biocompatibility of FeO/MoS_2_-BSA nanocomposites.

### Magnetic Resonance Imaging of FeO/MoS_2_-BSA in vitro and in vivo

**Fig. 6:**
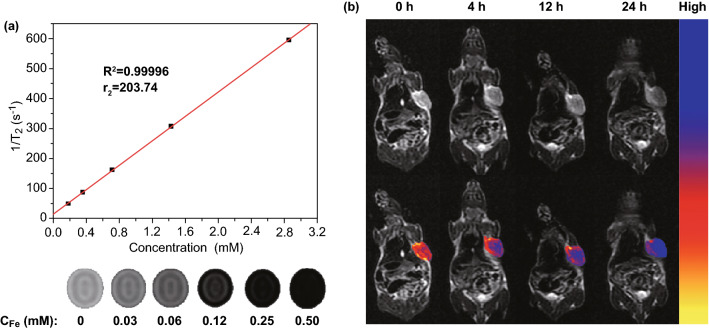
**a** *T*_2_-weighted MR images and *T*_2_ relaxation rates (*r*_2_) of FeO/MoS_2_-BSA nanocomposites. **b** *Magnetic* resonance *imaging* of *tumor* bearing mice and pseudo color *imaging* of *tumor* after tail vein injection for 0, 4, 12, and 24 h in vivo

Owing to the good paramagnetic behavior of FeO nanoparticles, FeO/MoS_2_-BSA nanocomposites are potential *T*_2_-weighted contrast agents for MRI. As shown in Fig. 6a, the signals of *T*_2_-weighted MRI are significantly enhanced as the increasing the concentration of Fe, and the transverse relaxivity value (*r*_2_) is calculated as 203.74 mM^−1^ s^−1^, which indicates that FeO/MoS_2_-BSA nanocomposites could act as promising *T*_2_-weighted MRI contrast agents. In order to further evaluate their feasibility as *T*_2_-weighted MRI contrast agents in vivo, the MRI of U14 *tumor* bearing mice were performed after intravenous injection with FeO/MoS_2_-BSA nanocomposites by 3.0 T human MRI scanner. As shown in Figs. 6b and S17, the *tumor* region is getting darker and lower signal intensity with the prolonging of injection time. The biodistribution of FeO/MoS_2_-BSA nanocomposites in vivo further demonstrated the good *tumor* accumulation of FeO/MoS_2_-BSA nanocomposites by enhanced permeability and retention (EPR) effect (Fig. S18). These results reveal that FeO/MoS_2_-BSA nanocomposites have good potential for serving as *T*_2_-weighted MRI contrast agents.

## Conclusions

In summary, the innovative FeO/MoS_2_-BSA nanocomposites were constructed to serve as “weapon” for highly efficient synergetic enhanced CDT/PTT of cancer. Not only the FeO nanoparticles and MoS_2_ nanosheets could generate the •OH individually as *Fenton* reagent and nanoenzyme, but also the co-catalytic effect between Mo^4+^ ions and Fe^3+^ ions is able to enhance *Fenton* reaction efficiency for more efficiency CDT. Importantly, FeO/MoS_2_-BSA nanocomposites shown excellent photothermal properties under irradiation with 1064 nm laser, achieving synergetic PT-enhanced CDT and PTT. The outstanding treatment effect has been illustrated both in vitro and in vivo. In addition, the good *magnetic* property enabled FeO/MoS_2_-BSA nanocomposites apply to the promising contrast agents for *T*_2_-weighted MRI, supplying accurate and clear information for *tumor* diagnosis. This collaborative strategy based on *NIR* II light-motivated photothermal effect and co-catalysis may provide a new idea for constructing effective *Fenton* nanoagents, and boost the development of CDT in future.

## Electronic supplementary material

Below is the link to the electronic supplementary material.

Supplementary file1 (PDF 1395 kb)
