# Supplementary material for: Boosting Chemodynamic Therapy by the Synergistic Effect of Co-Catalyze and Photothermal Effect Triggered by the Second Near-Infrared Light
Source: Nanomicro Lett. 2020 Sep 2;12:180. doi: 10.1007/s40820-020-00516-z (PMC7770794; doi:10.1007/s40820-020-00516-z)
Supplement: Supplementary file 1 — Supplementary file1 (PDF 1395 kb) [file 40820_2020_516_MOESM1_ESM.pdf]

Supporting Information for

Boosting Chemodynamic Therapy by the Synergistic Effect of Co-Catalyze and Photothermal Effect Triggered by the Second Near-Infrared Light

Songtao Zhang[1, 2], Longhai Jin[4], Jianhua Liu[4], Yang Liu[1], Tianqi Zhang[4], Ying Zhao[1], Na Yin[1], Rui Niu[1], Xiaoqing Li[1], Dongzhi Xue[1], Shuyan Song[1], Yinghui Wang[1, *], Hongjie Zhang[1, 2, 3, *]

[1]State Key Laboratory of Rare Earth Resource Utilization, Changchun Institute of Applied Chemistry (CIAC), Chinese Academy of Sciences (CAS), Changchun, 130022, People's Republic of China

[2]University of Chinese Academy of Sciences, Beijing, 100049, People's Republic of China

[3]Department of Chemistry, Tsinghua University, Beijing, 100084, People's Republic of China

[4]Department of Radiology, The Second Hospital of Jilin University, Changchun, 130041, People's Republic of China

*Corresponding authors. E-mail: yhwang@ciac.ac.cn (Yinghui Wang), hongjie@ciac.ac.cn (Hongjie Zhang)

S1 Measurement of Photothermal Conversion Efficiency of FeO/MoS_2-BSA Nanocomposites in 1064 nm Laser Irradiation

The photothermal conversion efficiency (η) of FeO/MoS_2-BSA nanocomposites is calculated by equations shown as follows [S1]:

$$\eta = [hS(T_{max} - T_{surr}) - Q_0] / [I(1 - 10^{-A1064})] \times 100\% \quad \text{(S1)}$$

$$hS = (\Sigma m_i C_{p,i})/\tau_s \quad \text{(S2)}$$

$$t = \tau_s \times (-\ln\theta) \quad \text{(S3)}$$

$$\theta = (T - T_{surr})/(T_{max} - T_{surr}) \quad \text{(S4)}$$

$$Q_0 = hS(T_{max, water} - T_{surr}) \quad \text{(S5)}$$

Where h is the heat transfer coefficient, S is the sample container surface area, T_{max} is the steady state maximum temperature, T_{surr} is the ambient room temperature, Q_0 is energy input of quartzsample cell and solvent without FeO/MoS_2-BSA nanocomposites, I is the laser power (0.75 W cm^{-2}), and A1064 is the absorbance of FeO/MoS_2-BSA nanocomposites at 1064 nm. The value of hS is calculated by Eq. S2, Where τ_s is the characteristic thermal time constant, the mass concentration of the FeO/MoS_2-BSA nanocomposites solution was 200 μg mL^{-1}, and its heat capacity (Cp) was approximated to be 4.2 J g^{-1} k^{-1} (the heat capacity of water).The heat energy (Q_0) of the quartzsample cell and solvent without FeO/MoS_2-BSA

nanocomposites solution was measured independently calculated by Eq. S5, Therefore according to Eqs. S3-S4, time constant (τ_s) is calculated to be =175.04 s obtained from linear-fitted plot of t vs –Lnθ (Fig. S11) after cooling. And based on the Eq. S2, the hs is calculated to be 4.8 mW °C^{-1}. Then the η can be calculated to be 56% by equations mentioned above.

S2 Supplementary Figures and Table

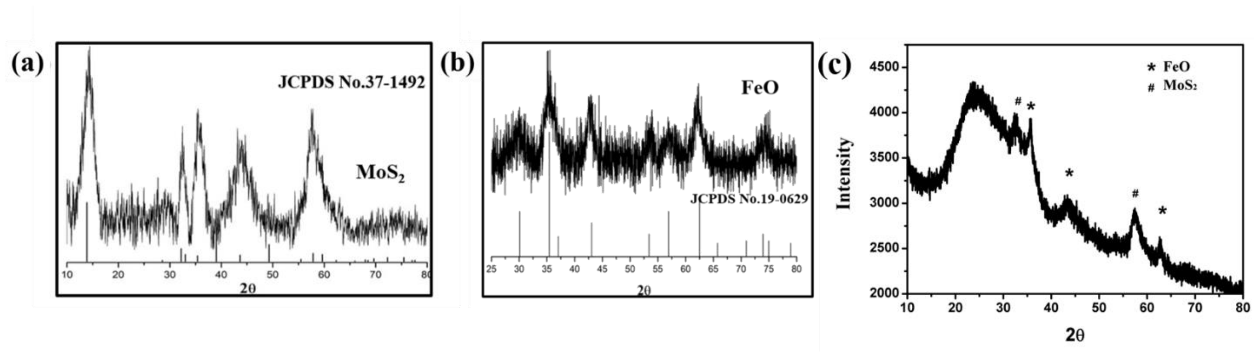

Fig. S1 XRD patterns of (**a**) MoS_2 nanosheets and (**b**) FeO nanoparticles and (**c**) FeO/ MoS_2-BSA nanocomposites

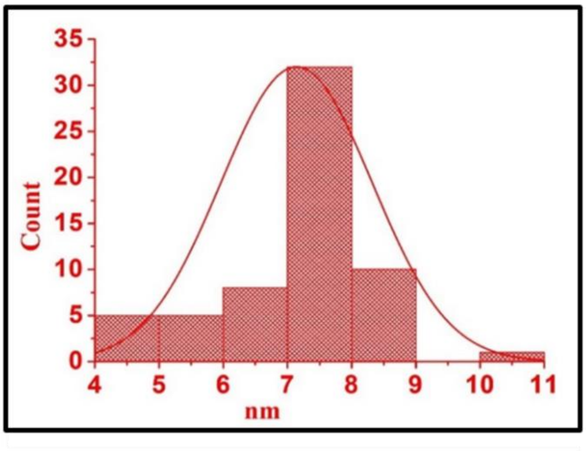

Fig. S2 Particle size analysis chart of FeO nanoparticles

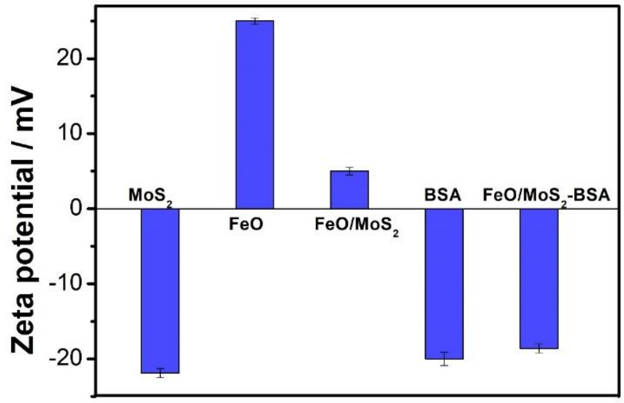

Fig. S3 Zeta potential of MoS_2, FeO, FeO/MoS_2, BSA, and FeO/MoS_2-BSA

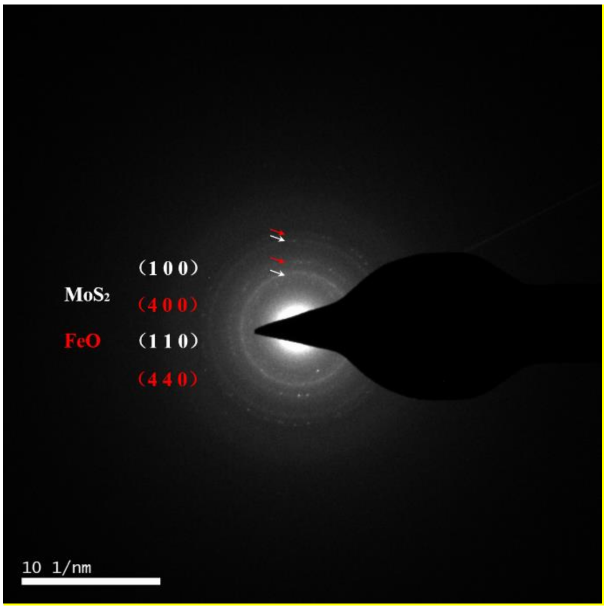

Fig. S4 SAED image of the FeO/MoS_2-BSA nanocomposites

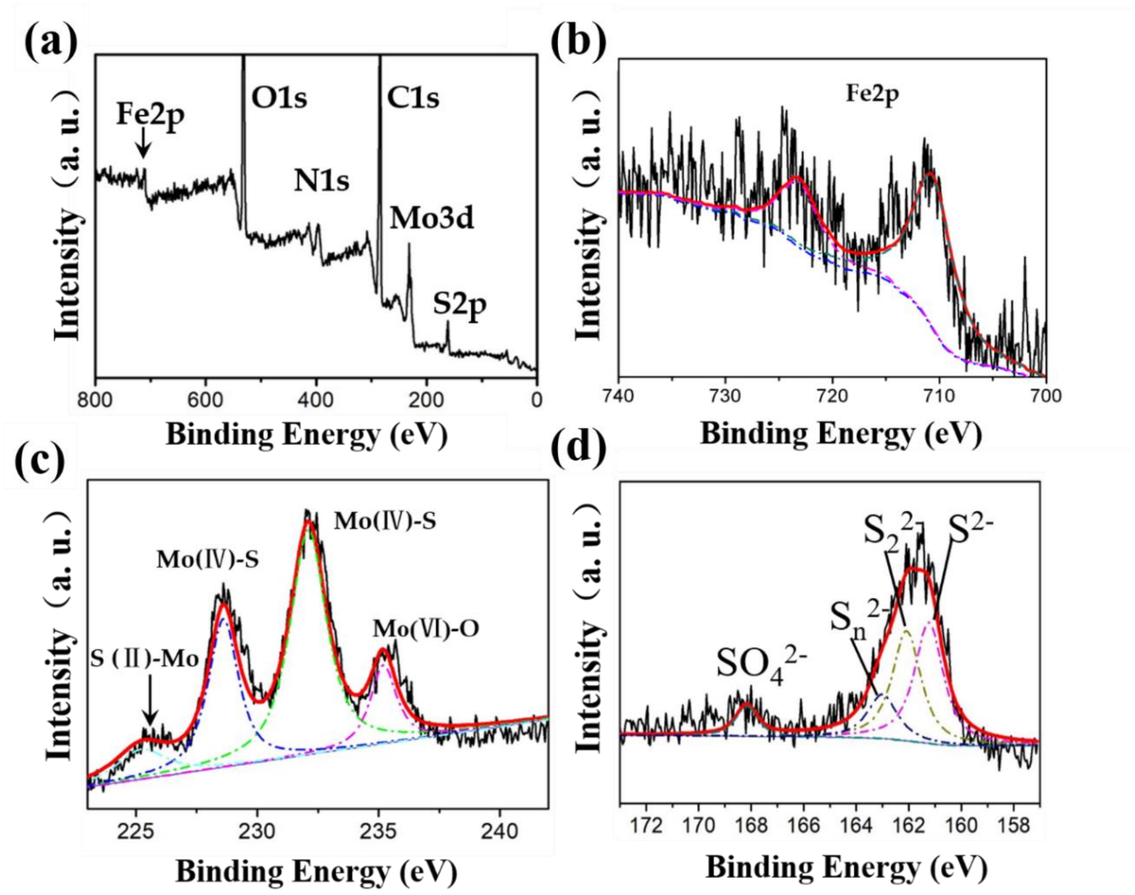

Fig. S5 (**a**) XPS survey spectrum of FeO/MoS_2-BSA nanocomposites. (**b-d**) Fe, Mo, S element XPS spectra, together with their corresponding fitting curves (the fitting curves were marked with the dash-dot lines)

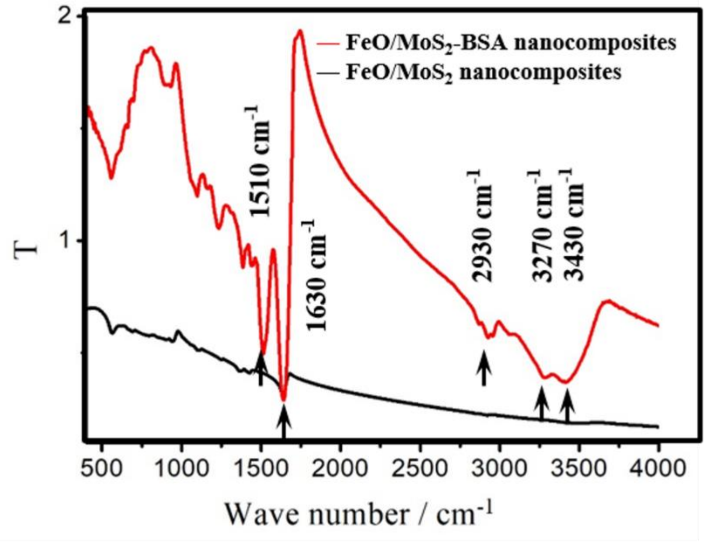

Fig. S6 Fourier transform infrared spectroscopy (FT-IR) of as-prepared FeO/MoS_2 nanocomposites and FeO/MoS_2-BSA nanocomposites

In Fig. S6, compared to the Fourier transform infrared spectroscopy (FT-IR) of FeO/MoS_2 nanocomposites, the characteristic absorption peaks only appeared in the infrared absorption spectrum of FeO/MoS_2-BSA nanocomposites at 1510, 1630, 3270, 2930, and 3430 cm^{-1} represent the absorption peak of C-N, C=O, N-H of amide bond, CH_2 and NH_2 respectively in BSA molecule, it indicated that the BSA molecule was modified on FeO/MoS_2 nanocomposites successfully.

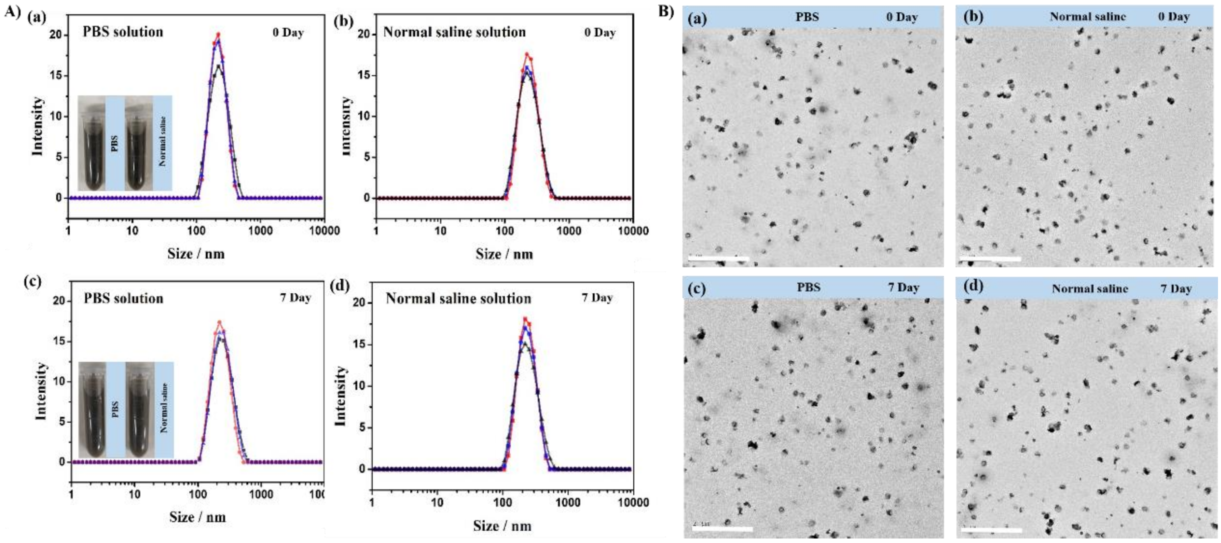

Fig. S7 (**A**) The pictures and hydrodynamic diameter of FeO/MoS_2-BSA nanocomposites dispersed in PBS and normal saline solution for 0 and 7 days. (**B**) TEM of FeO/MoS_2-BSA nanocomposites dispersed in PBS and normal saline solution for 0 and 7 days (scale bar: 2 μm)

Table S1 Hydrodynamic diameter, average ± SD of the HD and PDI of FeO/MoS_2-BSA dispersed in PBS and normal saline solution (n = 3)

Solution	Number	HD (nm)	+ SD	PDI
**PBS**	1	234.6	77.6	0.087
	2	222.8	59.4	0.062
	3	224.0	62.0	0.084
**Normal saline**	1	247.1	84.8	0.113
	2	245.3	75.6	0.098
	3	245.6	88.5	0.147

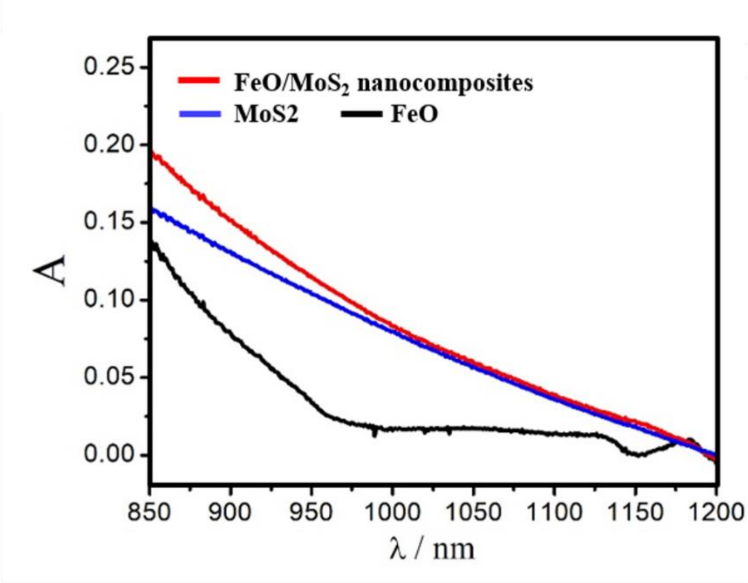

Fig. S8 UV-vis absorption spectra of FeO/MoS_2-BSA nanocomposites, MoS_2 nanosheets and FeO nanoparticle

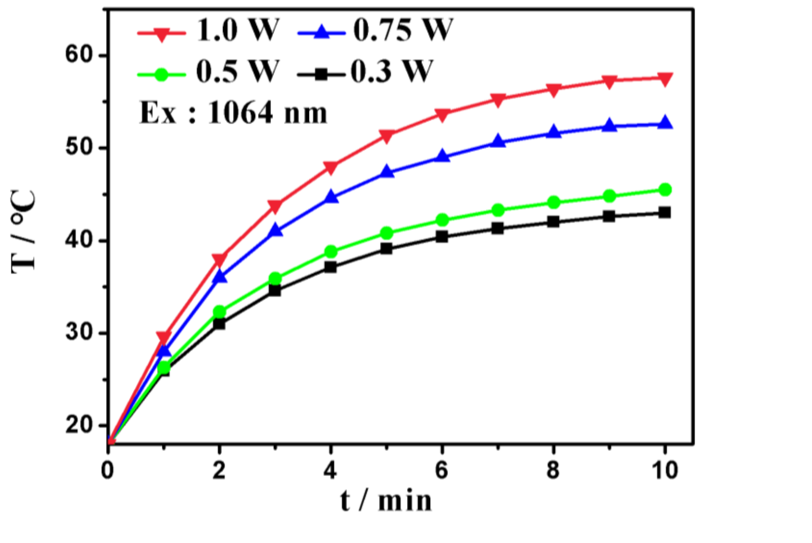

Fig. S9 Photothermal heating curves of FeO/MoS_2-BSA nanocomposites under the 1064 nm laser irradiation with different laser power

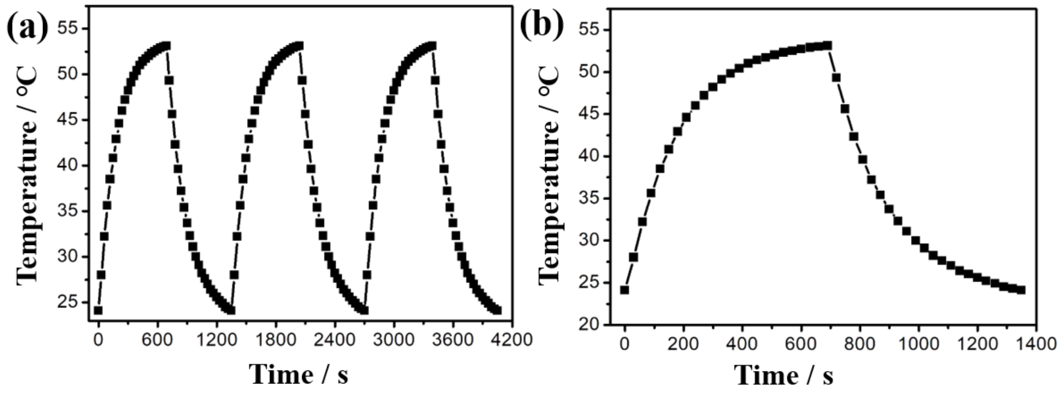

Fig. S10 (a, b) The temperature curves of FeO/MoS_2-BSA nanocomposites solution (200 µg mL^{-1}) under the 1064 nm laser irradiation (0.75 w cm^{-2}) with time

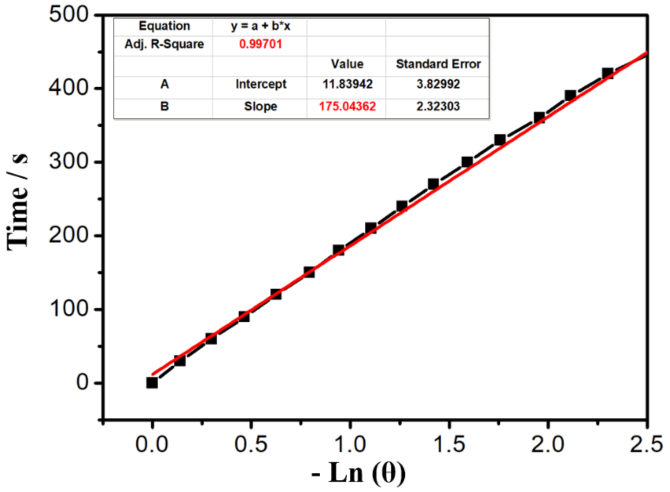

Fig. S11 Plot of cooling time after 10 min versus negative natural logarithm of driving force temperature (the linear fitted curves were marked with the red line with τ_s =175.04 s)

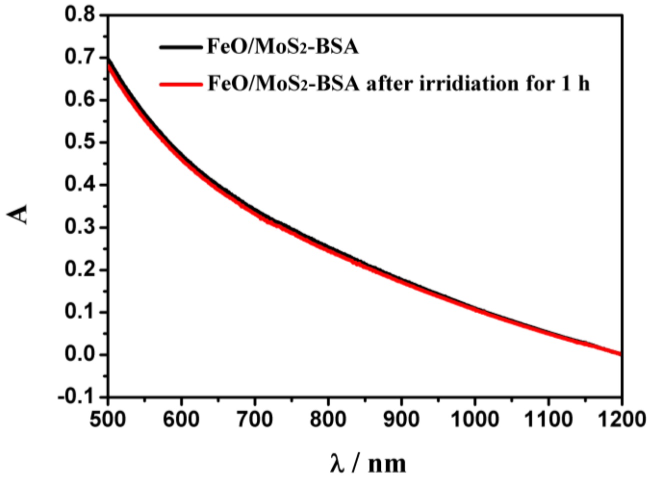

Fig. S12 UV-vis absorption spectra of FeO/MoS_2-BSA under 1064 nm laser irradiation (0.75 w cm^{-2}) for 0 and 1 h

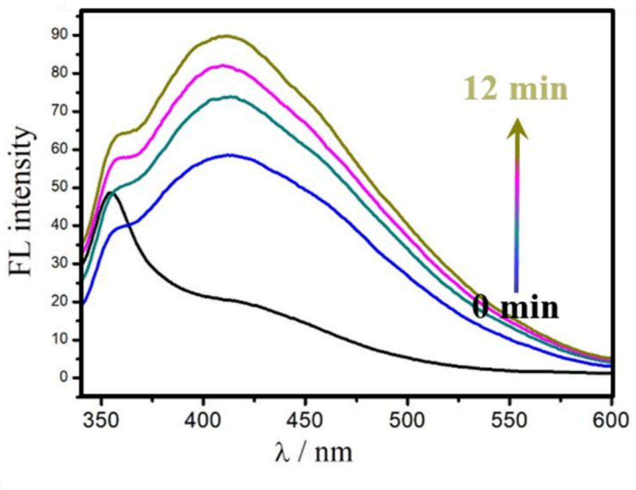

Fig. S13 Fluorescence spectrums of p-phthalic acid (PTA) mixed with H_2O_2 and FeO/MoS_2-BSA nanocomposites with time changed from 0 min to 12 min under the 1064 nm laser irradiation

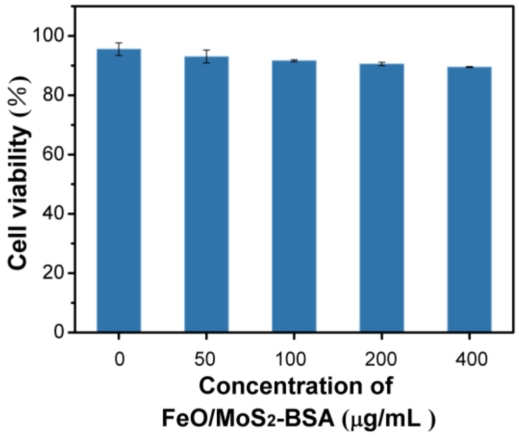

Fig. S14 Cell viability of Hela cells with different concentration of FeO/MoS_2-BSA (Data are means ± SD; N = 3)

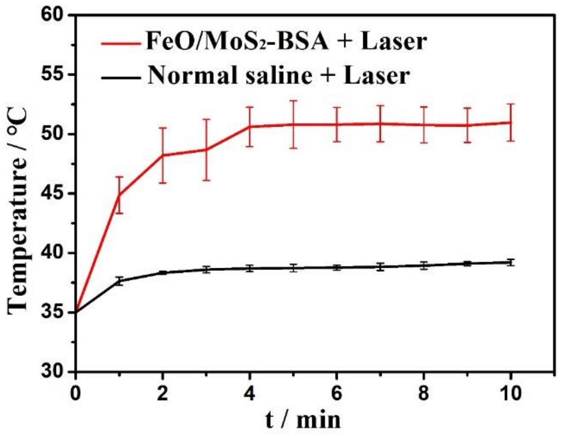

Fig. S15 Irradiation-time-dependent temperature changes of tumor-bearing mice under 1064 nm irradiation with or without injection of FeO/MoS_2-BSA

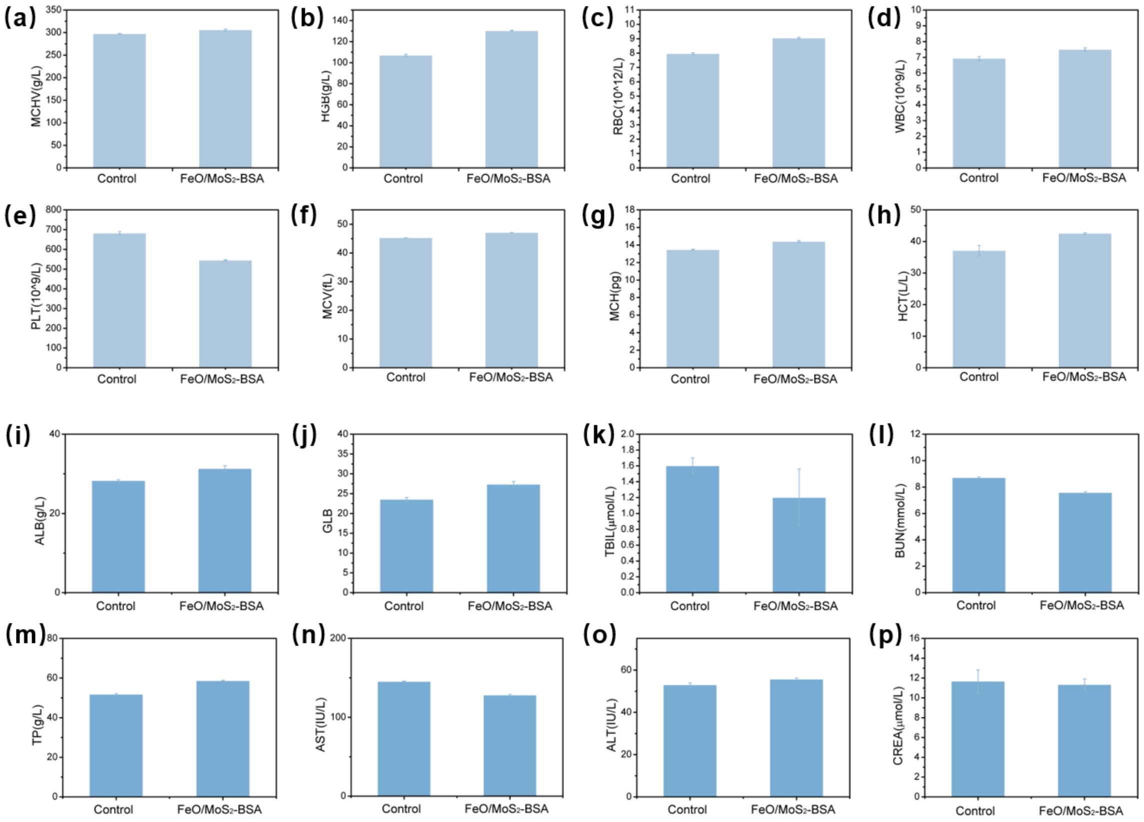

Fig. S16 Blood analysis. (**a-h**) Hematology analysis detected by the complete blood after intravenous injection of FeO/MoS_2-BSA at 30 d. (**i-p**) Blood biochemistry detection by blood serum after intravenous injection of FeO/MoS_2-BSA at 30 d

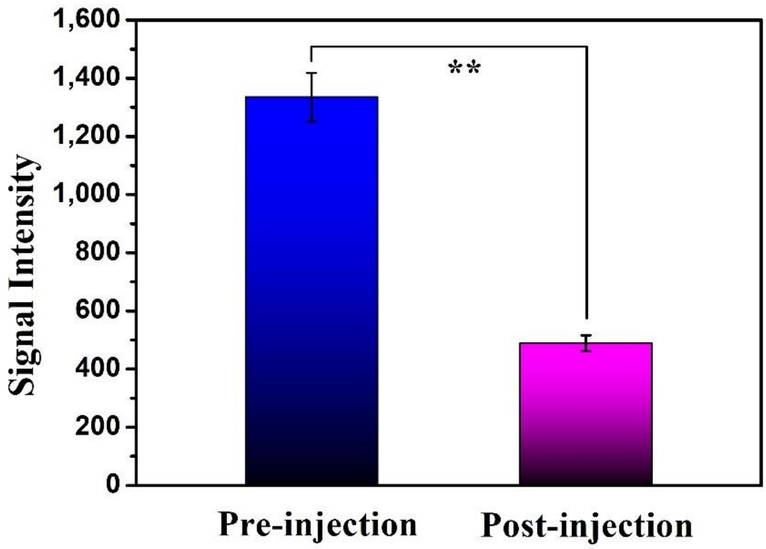

Fig. S17 Quantification of MR signals in tumors after intravenous injection of FeO/MoS_2-BSA for 24 h *in vivo* (**$P < 0.01$)

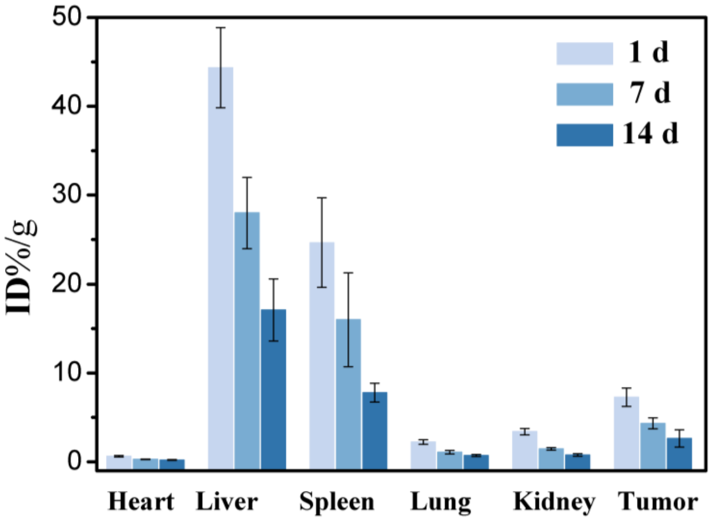

Fig. S18 Biodistributions of Mo atom in tumor and main organs at different time (1, 7, and 14 days)

Supplementary Reference

[S1]J. Yu, W. Yin, X. Zheng, G. Tian, X. Zhang et al., Smart MoS_2/Fe_3O_4 nanotheranostic for magnetically targeted photothermal therapy guided by magnetic resonance/photoacoustic imaging. Theranostics **5**(9), 931-945 (2015). https://doi.org/10.7150/thno.11802
